# Two-Step Synthesis and Hydrolysis of Cyclic di-AMP in *Mycobacterium tuberculosis*

**DOI:** 10.1371/journal.pone.0086096

**Published:** 2014-01-23

**Authors:** Kasi Manikandan, Varatharajan Sabareesh, Nirpendra Singh, Kashyap Saigal, Undine Mechold, Krishna Murari Sinha

**Affiliations:** 1 Institute of Molecular Medicine, New Delhi, India; 2 Council of Scientific and Industrial Research - Institute of Genomics and Integrative Biology, Delhi and IGIB Extension Centre (Naraina), New Delhi, India; 3 Central Instrument Facility, University of Delhi South Campus, New Delhi, India; 4 Institut Pasteur, CNRS UMR 3528, Unité de Biochimie des Interactions macromoléculaires, Paris, France; Indian Institute of Science, India

## Abstract

Cyclic di-AMP is a recently discovered signaling molecule which regulates various aspects of bacterial physiology and virulence. Here we report the characterization of c-di-AMP synthesizing and hydrolyzing proteins from *Mycobacterium tuberculosis*. Recombinant Rv3586 (MtbDisA) can synthesize c-di-AMP from ATP through the diadenylate cyclase activity. Detailed biochemical characterization of the protein revealed that the diadenylate cyclase (DAC) activity is allosterically regulated by ATP. We have identified the intermediates of the DAC reaction and propose a two-step synthesis of c-di-AMP from ATP/ADP. MtbDisA also possesses ATPase activity which is suppressed in the presence of the DAC activity. Investigations by liquid chromatography -electrospray ionization mass spectrometry have detected multimeric forms of c-di-AMP which have implications for the regulation of c-di-AMP cellular concentration and various pathways regulated by the dinucleotide. We have identified Rv2837c (MtbPDE) to have c-di-AMP specific phosphodiesterase activity. It hydrolyzes c-di-AMP to 5′-AMP in two steps. First, it linearizes c-di-AMP into pApA which is further hydrolyzed to 5′-AMP. MtbPDE is novel compared to c-di-AMP specific phosphodiesterase, YybT (or GdpP) in being a soluble protein and hydrolyzing c-di-AMP to 5′-AMP. Our results suggest that the cellular concentration of c-di-AMP can be regulated by ATP concentration as well as the hydrolysis by MtbPDE.

## Introduction

Bacteria have to adapt to changing conditions during their life cycle. They have developed different pathways to sense these changes and signal them accordingly for adaptation. Two-component signal transduction system (TCS) [1], extracellular and intracellular small signaling molecules play important roles in regulating expression of a series of genes for bacterial adaptation [2]. Autoinducers like acyl homoserine lactones [3] and modified oligopeptides [4] are extracellular signaling molecules used by both Gram positive and negative bacteria for cell-cell communication (quorum sensing). Intracellular signaling molecules act as second messengers and respond to different types of stimuli and hence offer flexibility to the cells. Cyclic AMP (cAMP) and ppGpp are two of the common signaling molecules present in bacteria. cAMP regulates the utilization of alternative carbon source [5] whereas ppGpp is involved in stringent response during nutrient starvation [6]. cAMP also regulates other cellular functions like flagellum biosynthesis, virulence, biofilm formation directly or indirectly whereas ppGpp is involved in quorum sensing, virulence etc [6], [7]. Cyclic-di-GMP (c-di-GMP) and c-di-AMP, two dinucleotides in prokaryotes act as secondary molecules and have regulatory roles in bacterial cell cycle, adaptation and virulence [6], [8]–[10]. Bacteria mostly use c-di-GMP to regulate switching from free living, planktonic mode to motile, biofilm lifestyle [11]. Recent work has shown that c-di-GMP regulates processes like synthesis of adhesins and exopolysaccharide matrix components, virulence, cell cycle [12]. Proteins which synthesize c-di-GMP from GTP contain additional domains like PAS, GAF, CHASE, BLUF which sense different environmental cues and signal is integrated in the c-di-GMP network [12]. Same is also true for c-di-GMP hydrolyzing proteins. Proteins encoding for these two activities are present across the prokaryotes implying conservation of these pathways in bacteria. c-di-AMP has been discovered recently in *Bacillus subtilis* [13]. DisA, a protein which scans the chromosome for DNA damage and forms focus at the break point was shown to synthesize c-di-AMP from ATP (diadenylate cyclase activity) [13]. The diadenylate cyclase (DAC) activity decreases in presence of branched chain DNA substrate implying a role of c-di-AMP in signaling DNA damage [10]. In *Bacillus subtilis*, DisA delays sporulation on encountering DNA damage which is signaled by a decrease in the cellular c-di-AMP level. c-di-AMP has also been found to regulate the biosynthesis of cell wall component in *Staphylococcus aureus* and *Bacillus subtilis* [8], [14], [15] and promotes virulence of *Listeria monocytogenes* [16].

*Mycobacterium tuberculosis* (*M*. *tb*.), one of the three most infectious pathogens worldwide goes through different stages in their life cycle. They reside within human macrophages in latent stage for long periods of time before they are reactivated and infect the host. They have to adapt to these conditions and signaling molecules and pathways assume special significance in the adaptation process [17]–[19]. Moreover, *M*. *tb*. also has to overcome the immune response produced by macrophages during their latent stage. Many of the agents which are produced as part of the immune response like reactive nitrogen and oxygen species cause different damages to the bacterial DNA. Signaling and repair of the DNA damage thus become important in mycobacterial pathophysiology [20].

Mycobacterium has a *disA* homolog (*rv3586* in H37Rv strain) which exists in an operon with *radA* (*rv3585*). Rv3586 has recently been shown to have DAC activity [21]. To understand the role of c-di-AMP in mycobacterial pathophysiology, we have undertaken the current work. Here we report the characterization of c-di-AMP synthesizing and hydrolyzing activities from mycobacterium. We show that the DAC activity of the recombinant Rv3586 is regulated by its own substrate, ATP and is also sensitive to the metal ion concentration. We have identified the intermediates of the DAC reaction and propose a two-step synthesis of c-di-AMP from ATP/ADP. By making mutant Rv3586 which does not have the DAC activity, we have shown that the protein possesses ATPase activity. We have identified Rv2837c as c-di-AMP specific phosphodiesterase. It hydrolyzes c-di-AMP in two steps: first to linear 5′-phospho diadenylate (pApA) which is further hydrolyzed to two molecules of 5′-AMP. We have named Rv3586 and Rv2837c as MtbDisA and MtbPDE respectively.

## Materials and Methods

### Purification of Recombinant MtbDisA and MtbPDE

The open reading frame (ORF) encoding MtbDisA was amplified from H37Rv genomic DNA using forward and reverse primers. The forward primer inserted an NdeI site at the initiation of ORF whereas the reverse primer introduced a SalI site 3′ to the termination codon. The insert (amplified product) was digested with NdeI and SalI and inserted between the same sites of pET28c (Novagen) to give the expression construct pDisA2. This construct expresses MtbDisA with an N-terminus His_6_ tag. The tagged DisA was expressed by transforming pDisA2 in Rosetta(DE3) *E*. *coli* cells. The expression of the protein was induced by growing the transformants in Luria broth at 37°C to A_600_≈0.5 followed by chilling the cells on ice for about an hour. Ethanol and isopropyl 1-thio-β-D-galactopyranoside (IPTG) were added to the culture to a final concentration of 2% and 0.2 mM respectively and the cells were further grown at 16°C for 18 hours. The cells were centrifuged and the pellet was used for the protein purification. The cell pellet was lysed by suspending in buffer A (lysis buffer) (50 mM Tris-HCl, pH 7.5, 0.25 M NaCl, 10% sucrose), 1 mg/ml lysozyme and 0.1% triton X-100 and incubated on ice for an hour (entire purification procedure was done in cold at 4°C unless otherwise mentioned). The lysate was sonicated to reduce viscosity, centrifuged to remove the insoluble material and the soluble extracts were applied to a Ni-NTA agarose (Qiagen) column pre-equilibrated with buffer A. The column was then washed with 5 column volume each of buffer B (50 mM Tris-HCl, pH 8.0, 0.25 M NaCl, 0.05% Triton X-100, 10% glycerol) and buffer B containing 50 mM imidazole. The bound proteins were then eluted with 3 column volume of buffer B containing 300 mM imidazole. The polypeptide composition of the fractions was monitored by SDS-PAGE. MtbDisA eluted in 300 mM imidazole fraction. The protein was dialyzed against buffer C containing 50 mm Tris-HCl, pH 8.0, 1 mm EDTA, 0.1% Triton X-100, 10% glycerol and 0.25 M NaCl. The dialysate was applied on DEAE-sephacel column pre-equilibrated with buffer C to remove the nucleic acid. The protein (MtbDisA) was recovered in flow through.The protein was further dialyzed against buffer B containing 100 mM NaCl for ≈12 hours and aliquoted and frozen at −80°C. 7.5 µg of the protein was analyzed on 12% SDS-PAGE (Figure S1B).

The mutant MtbDisA protein was generated by mutating D72 and G73 residues to alanine to give the mutant protein D72AG73A. The mutation was generated in the wild type *mtbdisA* gene in the construct pDisA2 using Quickchange site-directed mutagenesis kit (Stratagene). The resultant construct containing the mutant protein was named pDisA3. The mutant protein was purified by transforming pDisA3 in Rosetta(DE3) *E*. *coli* cells and following exactly the same procedure as that for the purification of the wild type protein.

The ORF of Rv2837c (MtbPDE) was PCR amplified from H37Rv genomic DNA. The forward primer inserted a BamHI site at the initiation whereas the reverse primer introduced an XhoI site 3′ to the termination codon. The insert (amplified product) was digested with BamHI and XhoI and inserted between similarly digested pET28-His_10_-Smt3 vector [22] to give the expression construct pPDE1. This construct expresses MtbPDE with an N-terminus His_10_-Smt3 tag. The protein was expressed by transforming pPDE1 in BL21(DE3) *E*. *coli* cells (Novagen). It was induced and purified following similar protocol as for MtbDisA. The tagged protein was eluted from Ni-NTA as before in buffer B containing 300 mM imidazole. The eluate was dialyzed against buffer C containing 50 mm Tris-HCl, pH 8.0, 1 mm EDTA, 0.1% Triton X-100, 10% glycerol and 0.25 M NaCl. The dialysate was applied on DEAE-sephacel column pre-equilibrated with buffer C to remove the nucleic acid. The protein (MtbPDE) was recovered in flow through and was mixed with Smt3-specific protease Ulp1 to cleave the His_10_-Smt3 tag (Ulp1:protein ratio was 1∶500). The protein was dialyzed for ∼12 hours (overnight) against buffer B containing 100 mM NaCl. The tag-free protein was recovered in flow-through by passage of the dialysate over Ni-NTA agarose column pre-equilibrated with buffer B containing 100 mM NaCl. The protein was aliquoted and frozen at −80°C. 7.5 µg of the protein was analyzed on 12% SDS-PAGE (Figure S1B).

Residues D130 and H131 of the wild type MtbPDE were mutated to alanine to give the mutant protein D130AH131A. The mutation was generated in a two step process using QuikChange site-directed mutagenesis kit (Stratagene). In the first step D130 was mutated to alanine using pPDE1 as template to give the construct pPDE2. pPDE2 was then used to mutate H131 to ‘A’ resulting in the construct pPDE3 which has both D130 and H131 mutated to alanine. pPDE3 expresses the mutant PDE protein, D130AH131A with a His_10_-Smt3 tag fused at its N-terminus. Recombinant D130AH131A was expressed and purified following the same procedure as for the wild type PDE.

### Assay for the Enzyme Activity of MtbDisA and MtbPDE

The diadenylate cyclase (DAC) activity of the purified MtbDisA was determined by doing the enzyme assay in a reaction mixture containing 25 mM Tris-HCl (pH 8.5), 25 mM NaCl, 0.6 mM MnCl_2_, 300 µM ATP and 1 µM of MtbDisA. The reaction mixture was incubated at 37°C for 15 min or as mentioned followed by terminating the reaction by adding 5 mM EDTA. The hydrolysis of c-di-AMP by MtbPDE was determined in 10 µl reaction mixtures containing 50 mM Tris-HCl (pH 8.0), 5 mM Mn^2+^, 1 mM DTT, 0.1 mM c-di-AMP, indicated amounts of MtbPDE and incubating the reaction mixtures at 37°C for 10 min. The reaction was quenched by adding 10 mM EDTA. The formation/hydrolysis of c-di-AMP was determined by separating the reaction mixtures by reverse phase liquid chromatography (LC) either on Acquity UPLC (Waters) or Ultimate 3000 UHPLC (Dionex). The amount of c-di-AMP formed/hydrolyzed was calculated from the peak area of the chromatograms by interpolating the values to a standard plot.

For all the LC runs, 10 mM ammonium acetate (NH_4_OAc; pH 5.2; solvent A) and methanol (solvent B) were used as the mobile phase [10]. Acclaim RSLC 120 C18 (2.2 µm, 120 Å, 2.1 mm×100 mm) was the column utilized on Ultimate 3000 UHPLC. The Ultimate 3000 UHPLC was connected to an electrospray ionization (ESI) mass spectrometer, LTQ Orbitrap XL (Thermo Scientific). The experiments requiring only UV detection were carried out on Acquity UPLC. For the experiments involving LC in conjunction with mass spectrometry (LC-ESI-MS), Ultimate 3000 UHPLC was utilized. Ultimate 3000 UHPLC was capable of UV detection as well.

Waters Aquity UPLC had photodiode array detector. The output signal was monitored and processed using Empower software. The UPLC BEH C18 100 mm×2.1 mm column having particle size of 1.7 µM was used. The separation was achieved with a gradient of 10 mM ammonium acetate (pH 5.2-mobile phase A) and methanol (mobile phase B).The flow rate of mobile phase was 0.3 mL/min. The gradient was set as time (min)/% solution A: 0/100,0.8/95,4.9/50 and 8/5. The column was equilibrated with 100% A for two min before next injection. Column temperature was maintained at 40°C and the detection was monitored at 260 nm. The reaction mixtures (DAC or PDE) were diluted 16 times and 5 µl was injected.

The flow rates utilized for acquiring LC-ESI-MS data were varied between 0.05 ml/min and 0.2 ml/min. A gradient elution method optimized to the duration of 20 min was followed for all the experiments (solvent B (methanol): 5%–60% in 10 mins, 60%–95% in 2 mins, hold at 95% for 2 mins, 95%–5% in 3 mins and hold at 5% for 3 mins). The LC-ESI-MS runs were carried out by conventional ESI using Ion Max source of LTQ Orbitrap. Typically, the spray voltage was set to about 5 kV and the resolution (R) of orbitrap was set at 30,000 for all data acquisitions. The other tune parameters were: sheath gas, varied in the range 15–30; auxiliary gas: 5–12, sweep gas: 4–8; capillary temperature: 275^0^C; capillary voltage: 35 V and tube lens: 110 V. Tandem mass spectral (LC-ESI-MS/MS) data acquisition was carried out following collision induced dissociation (CID) accomplished within LTQ using helium as the collision gas. The normalized collision energy was set to 35 and in some experiments it was varied. The other parameters for the LC-ESI-MS/MS data recording were: precursor ion isolation width *m/z* 1.5, activation q 0.25, activation time 30 milliseconds. All the LC-ESI-MS and LC-ESI-MS/MS data were processed using Excalibur (Thermo Scientific).

### Matrix Assisted Laser Desorption/Ionization (MALDI) MS Characterization

#### Desalting of sample

The samples were mixed with 50 mM ammonium acetate and desalted using zip-tip C18 (Millipore,USA). In brief, C18 zip-tip was washed with 100% acetonitrile (ACN) and equilibrated with 0.1% trifluoroacetic acid (TFA). The samples were passed through the zip-tip 5 times for binding with the C18 column. The column was washed with 0.1% TFA to remove salt and the samples were eluted from the tip using 50% ACN in 5 µl.

The desalted samples were mixed with α-cyano 4-hydroxycinnamic acid (CHCA) in equal ratio and spotted on 384 well target plate. A 4800 Plus MALDI TOF/TOF™ (AB Sciex) mass spectrometer equipped with 337 nm laser operating in reflectron positive ion mode was utilized for all data acquisitions. External calibration was performed for all the spectra. The MS/MS experiments were carried out following CID utilizing air as the collision gas.

## Results

### DAC Activity of MtbDisA is Regulated by ATP

Rv3586 consists of an ORF of 358 amino acid residues. The region at N-terminus (amino acid residues 1–145) is the globular nucleotide-binding region which participates in the DAC reaction (Figure S1A) and has conserved amino acid residues at its active site [21]. MtbDisA has conserved ‘DGA’ (residue 72–74) and ‘RHR’ (residues 105–107) residues which interact with the adenine base and ribose sugar of c-di-AMP present at the nucleotide binding site [13].

**Figure 1:**
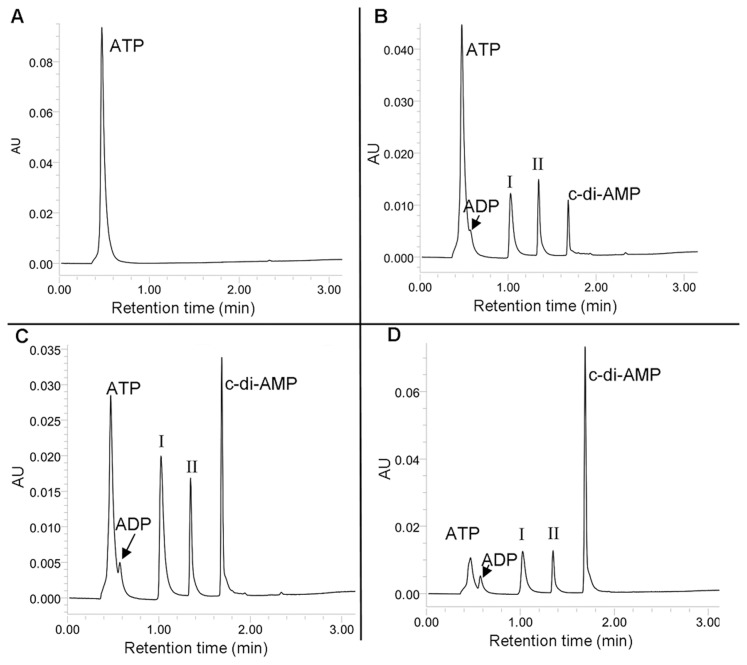
MtbDisA synthesizes c-di-AMP through two intermediates. DAC assay was done in 10 µl reaction mixtures containing 25 mM Tris-HCl (pH 8.5), 0.6 mM MnCl_2_, 300 µM ATP and 2 µM of MtbDisA and incubated at 37°C. The reaction mixture was subjected to reverse phase LC and the products were detected with UV (λ 260 nm). The absorption chromatograms with peaks labeled with the corresponding species have been shown. (A) Peak for only ATP is detected in the control reaction (without enzyme). ATP is converted into c-di-AMP in the presence of MtbDisA as can be seen in (B). Two more peaks are detected in ‘B’ which are the intermediates of the reaction and are labeled ‘I’ and ‘II’. In (B) the DAC reaction was terminated after 5 min whereas in (C) and (D) the reaction was continued for 10 and 60 min respectively. Peak for ADP has been labeled.

To study the enzymatic properties of MtbDisA, recombinant N-terminus His_6_-fusion protein was purified from *E*. *coli* (Figure S1B). The DAC activity of the protein was determined by doing the enzyme assay and characterizing the products by reverse phase LC [10] using NH_4_OAc and MeOH as mobile phase (Figure 1). The c-di-AMP in the reaction mixture was identified by comparing the retention time of the product with the commercially available c-di-AMP (BioLog). In addition, it was also confirmed by MALDI-MS/MS (data not shown) and LC-ESI-MS/MS (Figure S2). The separation of the reaction mixtures by reverse phase LC revealed the presence of products in addition to c-di-AMP as suggested by the appearance of additional peaks. Two of the distinct peaks which were observed were numbered ‘I’ and ‘II’ (Figure 1B). There is a distinct possibility that these peaks are the intermediates of the DAC reaction.

Peaks of ADP are also visible in the chromatograms (Figure 1B, C & D) and the amount of ADP formed remains around 100–150 pmoles during the course of the reaction and does not increase with time unlike the products of the DAC reaction.

**Figure 2:**
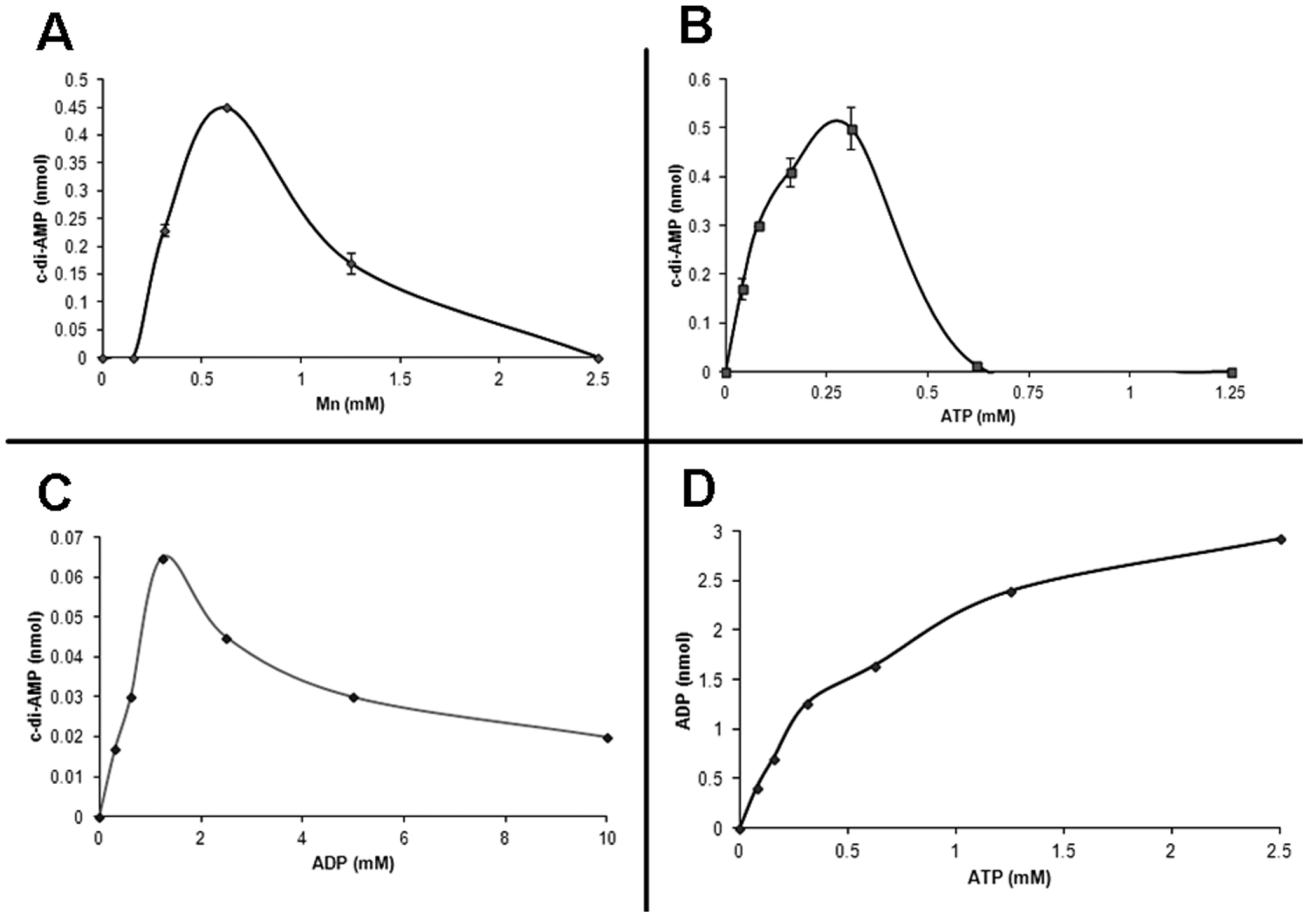
DAC activity is regulated by ATP. (A) Optimum Mn^2+^ concentration. The optimum concentration of Mn^2+^ was determined by doing the assay (20 µl) in presence of 25 mM Tris-HCl (8.5), 25 mM NaCl, 300 µM ATP, 1 µM of MtbDisA, varying concentrations of MnCl_2_ and the reaction was incubated at 37°C for 15 min. The plot shows the amount (nmoles) of c-di-AMP formed during the reaction as a function of Mn^2+^ concentration (B) ATP dependence. The extent of formation of c-di-AMP in 20 µl reaction mixtures was determined by doing the DAC assay in presence of 25 mM Tris-HCl (8.5), 0.6 mM Mn^2+^, 25 mM NaCl, 1 µM of MtbDisA and different concentrations of ATP. The reaction mixtures were incubated at 37°C for 15 min. The plot shows the formation of c-di-AMP as a function of ATP concentration. The data plotted in (A) and (B) represent the mean of three independent experiments ± standard deviation. (C) ADP inhibition. The DAC assay was carried out in 10 µl reaction mixtures containing 25 mM Tris-HCl (pH 8.5), 0.6 mM MnCl_2_, increasing concentrations of ADP as indicated, 2 µM of MtbDisA and incubated for 15 min at 37°C. The reaction mixtures were processed and analyzed as before. The plot shows the formation of c-di-AMP as a function of ADP concentration. (D) D72AG73A has ATPase activity. The DAC assay was carried out in 10 µl reaction mixtures containing 25 mM Tris-HCl (pH 8.5), 0.6 mM MnCl_2_, indicated concentrations of ATP, 2 µM of D72AG73A and incubated for 30 min at 37°C. The reaction mixtures were processed and analyzed as before. The plot shows the formation of ADP as a function of ATP concentration.

We tested different divalent metal ion co-factor for the DAC activity. The enzyme gives the optimum activity in the presence of Mn^2+^ (400 pmoles of c-di-AMP formed) whereas there is no significant activity in the presence of other bivalent metal ions tested (data not shown). The titration of Mn^2+^ ion revealed that the activity is very sensitive to the variation in the concentration of the metal ion. 450 pmoles of c-di-AMP is formed at 0.625 mM Mn^2+^ concentration and there is a decrease in the activity with any change in the concentration (Figure 2A).

We have made the interesting observation that MtbDisA is inhibited at higher concentrations of ATP (Figure 2B). The activity of the enzyme increases initially with the increase in the ATP concentration till about 0.3 mM and then it starts decreasing and becomes almost undetectable at 1 mM ATP which is the cellular concentration in mycobacterium. To confirm that the decrease in the activity is because of an increase in the ATP concentration and not depletion of metal ion by ATP, we titrated the Mn^2+^ concentration keeping the ATP concentration fixed at 0.6 mM but it did not increase the formation of c-di-AMP (data not shown). We speculate that this inhibition of the DAC activity by ATP has significance to mycobacterial physiology and will elaborate on it in discussion. A titration of ADP, the other substrate for the DAC reaction [21] revealed that like ATP, higher concentrations of ADP also inhibit the DAC activity of MtbDisA (Figure 2C) though the concentration of ADP giving the optimum activity is 1.2 mM.

Role of conserved amino acid residues ‘DG’ in the DAC assay was probed by mutating both the residues to alanine to give the mutant protein D72AG73A. D72AG73A did not catalyze the formation of c-di-AMP (data not shown). But it was found to have ATPase activity and hydrolyzed ATP to ADP. The ATPase activity is dependent on the ATP concentration (Figure 2D) and is not inhibited at higher concentrations of ATP unlike the DAC activity of MtbDisA.

### Reaction Intermediates Suggest a Two-step Synthesis of c-di-AMP

**Figure 3:**
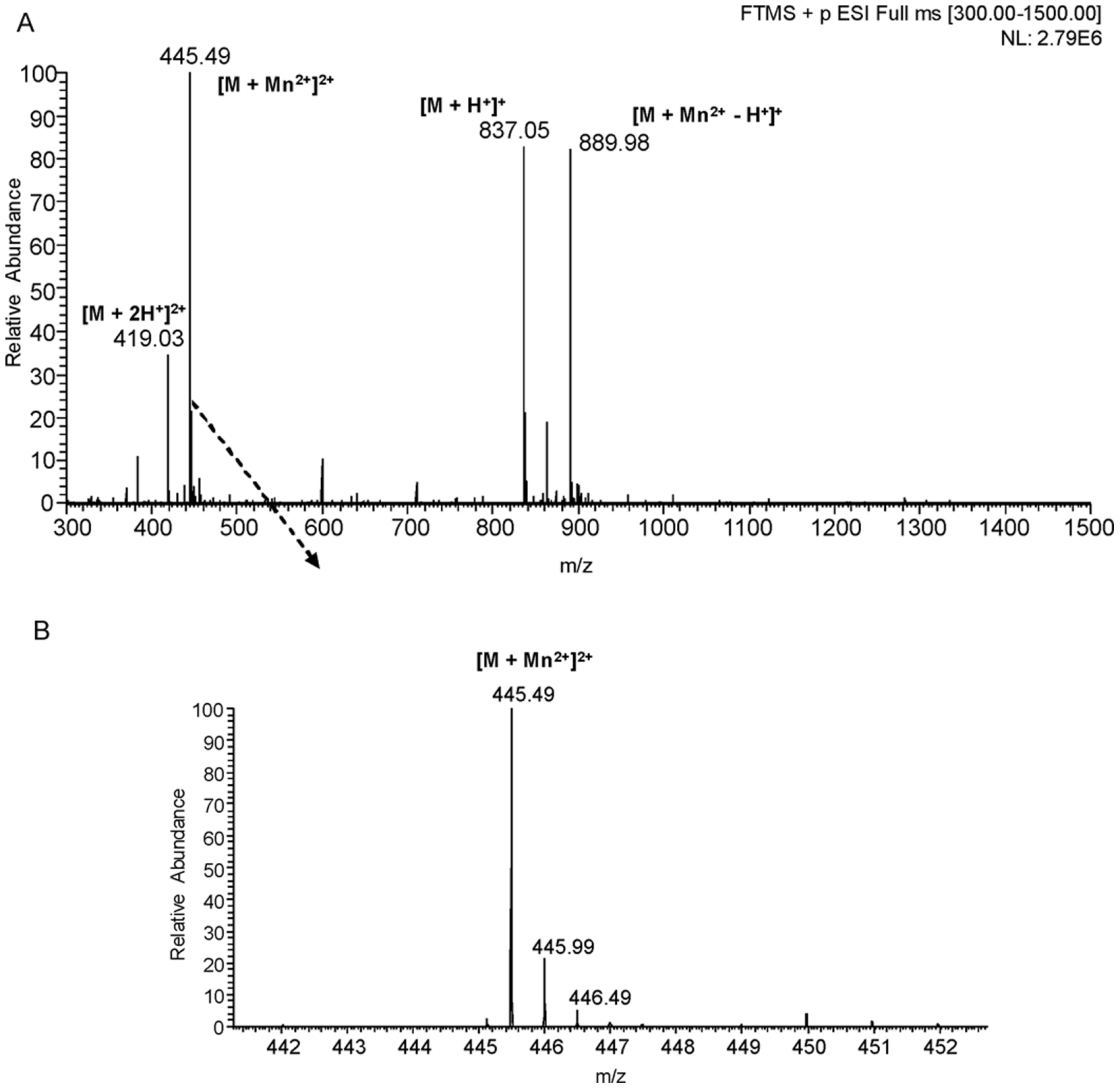
Detection of the intermediate ‘I’ of the DAC assay. (A) LC - ESI -MS spectrum showing the peaks corresponding to proton and manganese ion adducts of the intermediate ‘I’ of molecular mass 836 Da. (B) Expansion of the region, m/z 442–452 of the spectrum shown in (A): m/z values and intensity distribution of isotope peaks indicating doubly charged manganese ion adduct of ‘I’.

**Figure 4:**
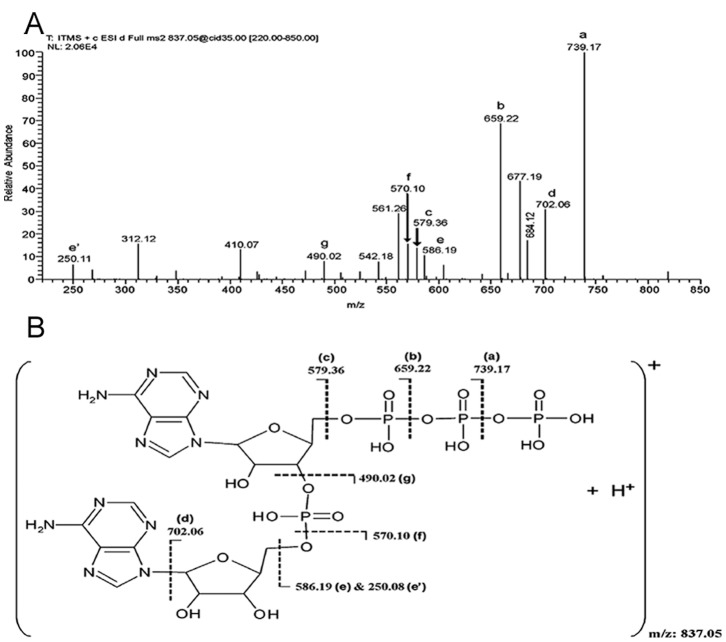
Reaction intermediate ‘I’ was determined to be pppApA. (A) LC-ESI-MS/MS spectrum of [M+H]^+^ precursor ion m/z 837.05 (reaction intermediate ‘I’). (B) Figure depicting the interpretation of the fragmentation as observed in (A).

The separation of the DAC reaction mixture by reverse phase LC coupled UV detection (λ 260 nm) showed the presence of two products (as suggested by the two very distinct peaks) which were numbered ‘I’ and ‘II’ (Figure 1B, C, D) in addition to c-di-AMP. We did time kinetics of the DAC reaction to find out whether the additional products were the intermediates of the DAC reaction. The concentrations of both ‘I’ and ‘II’ as well as that of c-di-AMP (reaction product) vary as the reaction progresses. After 5 min of incubation of the reaction mixture, ‘I’, ‘II’ and c-di-AMP can be detected (Figure 1B) and their amount increases further after 10 min of incubation (Figure 1C). After 60 min of incubation, the concentrations of ATP, ‘I’ and ‘II’ decrease considerably with concomitant increase in c-di-AMP concentration. This implies that ‘I’ and ‘II’ are the intermediates of the DAC reaction and c-di-AMP is finally produced from them. LC-ESI mass spectrum of the DAC reaction mixture determined the molecular masses of the intermediates ‘I’ and ‘II’ to be 836 and 890 Da respectively (Figure 3). The peaks at m/z 837.05 and 419.05 are due to singly ([M+H]^+^) and doubly protonated ([M+2H]^2+^) species of the intermediate ‘I’. LC-ESI-MS/MS and MALDI-MS/MS (data not shown) identified ‘I’ to be a linear 5′- triphosphate diadenylate (pppApA) (Figure 4).

**Figure 5:**
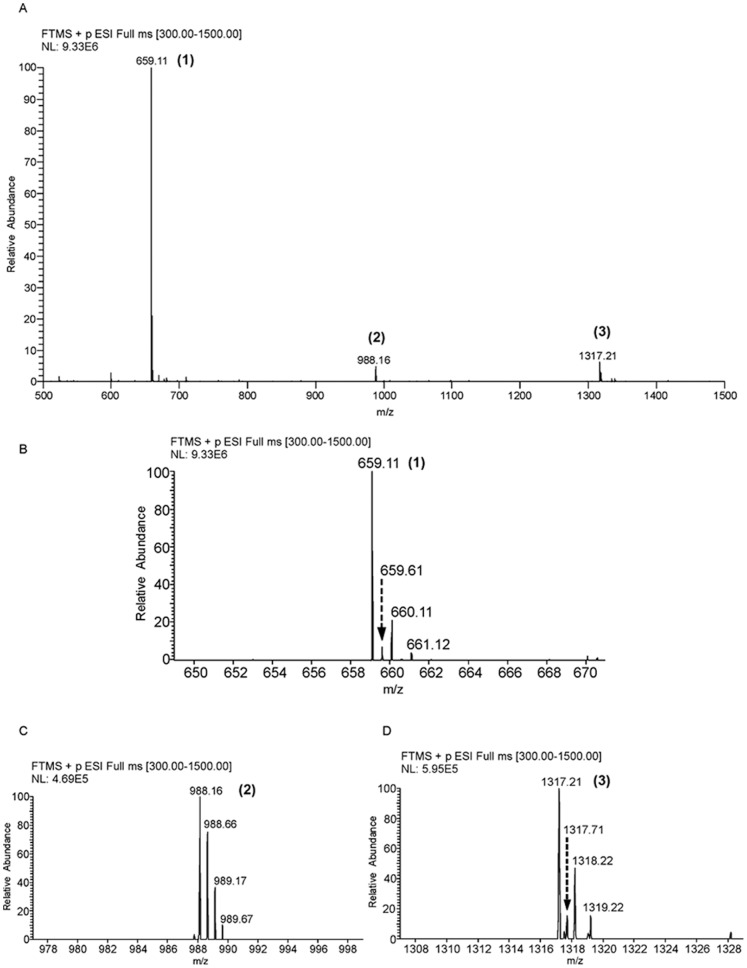
Two-step synthesis of c-di-AMP. The figure shows the formation of c-di-AMP from the two intermediates pppApA and ppApA. pppApA is first formed by conjugation between two molecules of ATP (step 1A). Two ADP molecules or one molecule of ADP and one ATP molecule can conjugate to give rise to ppApA (step 2A). pppApA and ppApA are then converted into c-di-AMP (steps 1B & 2B) through their respective Mn^2+^-adduct (transition state complex) as shown.

The signals at m/z 889.98 and 445.49 (Figure 3B) were ascertained to be singly [(M+Mn^2+^ – H^+^)^+^] and doubly charged [(M+Mn^2+^)] manganese adduct of the intermediate ‘I’, which is the intermediate ‘II’ (Figure S3). Signals corresponding to ADP was also detected by MALDI-MS and LC-ESI-MS and was confirmed by MS/MS. Additionally, MALDI mass spectrometric characterization of the DAC reaction mixture revealed the presence of another product, evident from a signal corresponding to a mass of 756 Da. MS/MS of precursor ion *m/z* 757 ([M+H]+) achieved by MALDI suggested this to be another linear dinucleotide involving conjugation of ADP and ATP/ADP giving rise to ppApA (Figure S4). Singly charged Mn^2+^-adduct of ppApA of molecular mass 810 Da (similar to the Mn^2+^-adduct of pppApA) was also detected by MALDI studies (data not shown). Based on these intermediates detected, we propose a two-step formation of c-di-AMP from ATP/ADP (Figure 5).

### c-di-AMP can Exist in Mutimeric Form

**Figure 6:**
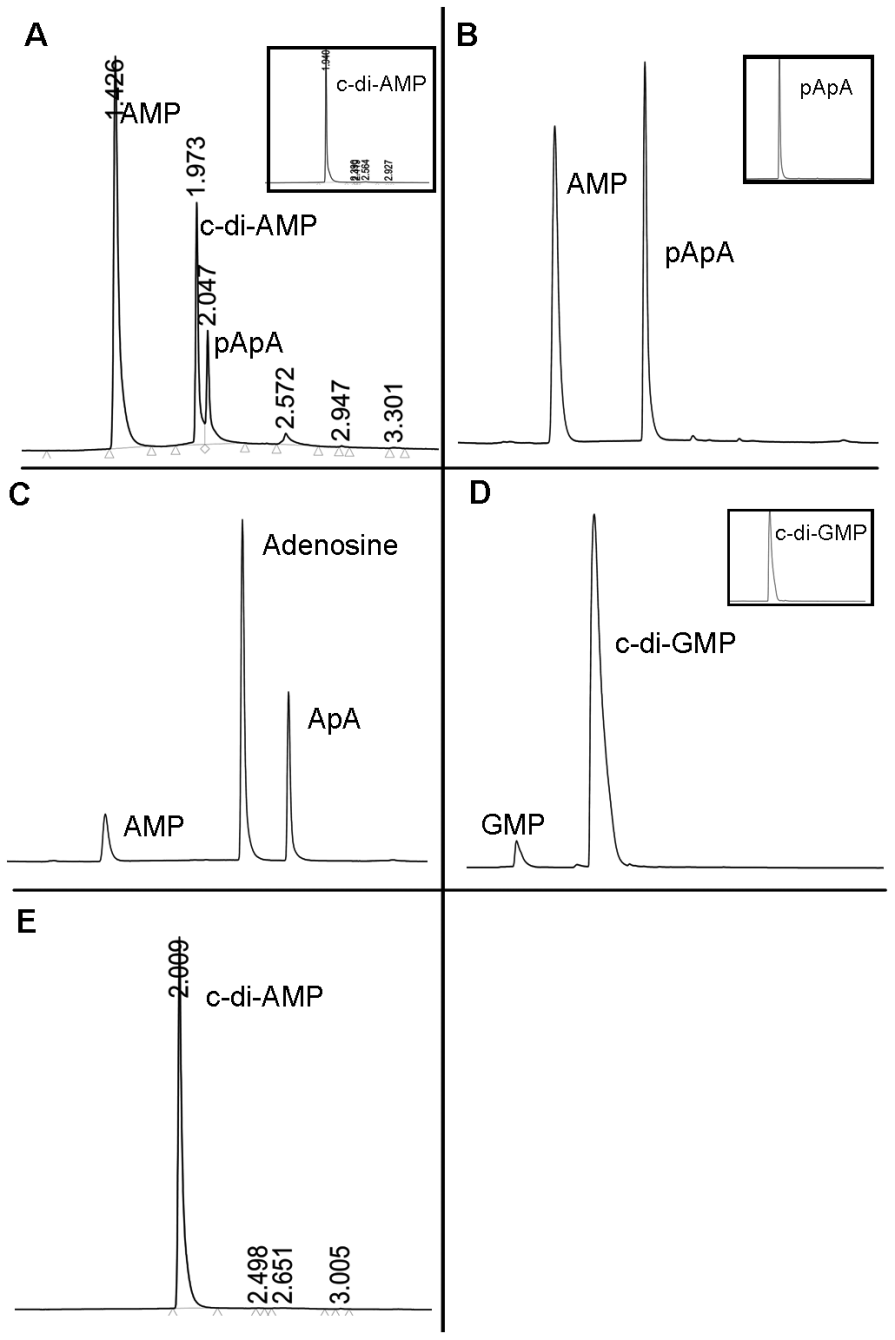
c-di-AMP can exist as multimers. The DAC reaction mixture was subjected to LC-ESI-MS and the mass spectra of the different multimeric forms have been shown here. (A) The LC-ESI mass spectrum showing signals corresponding to monomer, labeled ‘1′ and other multimeric forms, labeled ‘2′ and ‘3′ (B) Expansion of the region, m/z 648–672 of the spectrum in ‘A’ depicting isotope peaks corresponding to peak 1, which indicate presence of monomer and dimer (C) Expansion of the region, m/z 976–1000 of the spectrum in ‘A’, which shows isotope signals corresponding to peak 2 providing evidence for the presence of trimer of c-di-AMP (D) Expansion of the region, m/z 1306–1330 of the spectrum in ‘A’, indicating the presence of dimeric and tetrameric forms of c-di-AMP.

Signals were detected corresponding to the higher mass values than the molecular mass of c-di-AMP in LC-ESI-MS experiments of the DAC assays. We surmised that the higher molecular mass signals might arise because of the formation of the multimers of c-di-AMP. We analyzed the signals corresponding to the higher mass values in the DAC reaction mixture. Figure 6A shows the mass spectral evidences of the various multimers of c-di-AMP. Examination of these peaks (*m/z* values) suggested multimers of c-di-AMP adopting different charge states. The peak at m/z 659.61 (Figure 6B) among the isotope peak clusters of m/z 659.11 (labeled 1 in Figure 6A) could be interpreted as doubly protonated form of dimer, [2M+2H]^2+^, where M = 658.11 Da (2M: 2×658.11 Da = 1316.22 Da). The signal at *m/z* 988.16 (labeled 2 in Figure 6A) and peaks corresponding to its isotopes at *m/z* 988.66, 989.17 and 989.67 (Figure 6C) indicate a doubly protonated form of trimer of c-di-AMP, [3M+2H]^2+^ (3M: 3×658.11 Da = 1974.33 Da). Figure 6D shows the isotope peaks corresponding to peak labeled 3, m/z 1317.21 in Figure 6A. The isotope peaks at *m/z* 1318.22 and 1319.22 points to the presence of dimer of c-di-AMP, [2M+H]^+^ (2M: 2×658.11 Da = 1316.22 Da). Further, detection of the peak at *m/z* 1317.72 suggests the presence of tetramer of c-di-AMP as well; *m/z* 1317.21 and 1317.72 together can be interpreted as doubly protonated tetramer [4M+2H]^2+^, where 4M: 4×658.11 Da = 2632.44 Da. To the best of our knowledge, this is the first study reporting on mass spectrometric detection of the multimers of c-di-AMP. The multimers might exist in the assay mixture and they are transferred to the gas phase without disruption of those interactions that are responsible for the multimers to remain intact and hence are detected. Alternatively, they can also be formed during the course of ESI. These multimers have significance in impacting the bacterial physiology as has been seen in the case of c-di-GMP [23].

### Identification of the gene Encoding c-di-AMP Hydrolyzing Activity

The cellular concentration of c-di-AMP has to be tightly regulated like any other signaling molecule. Along with the regulation of the expression of the DisA protein, it also suggests the presence of a protein which can hydrolyze c-di-AMP. YybT is the only protein which has been shown to have c-di-AMP hydrolyzing activity [24]. Search of the mycobacterial genome database using YybT sequence did not show the presence of any such homolog. Even a search for the proteins containing COG3887, a group spanning the entire reading frame of YybT and which has been predicted to be present in signaling proteins containing modified GGDEF and DHH domain, did not identify any protein. Further screening of the database for proteins containing DHH/DHHA1 domain, domains present in YybT revealed Rv2837c to be the only protein containing the domains (Figure S1A). The ORF of Rv2837c contains 336 amino acid residues whereas YybT contains 659 residues. YybT is a membrane protein and about 300 amino acid residues at its N-terminus consists of a transmembrane, a PAS and ‘GGDEF’ domain which are absent in Rv2837c. Rv2837c contains DHH/DHHA1 domains which are located towards the C-terminus of YybT. DHH/DHHA1 has been suggested to be involved to hydrolyzing c-di-AMP in YybT [24].

### MtbPDE Hydrolyzes c-di-AMP to AMP

**Figure 7:**
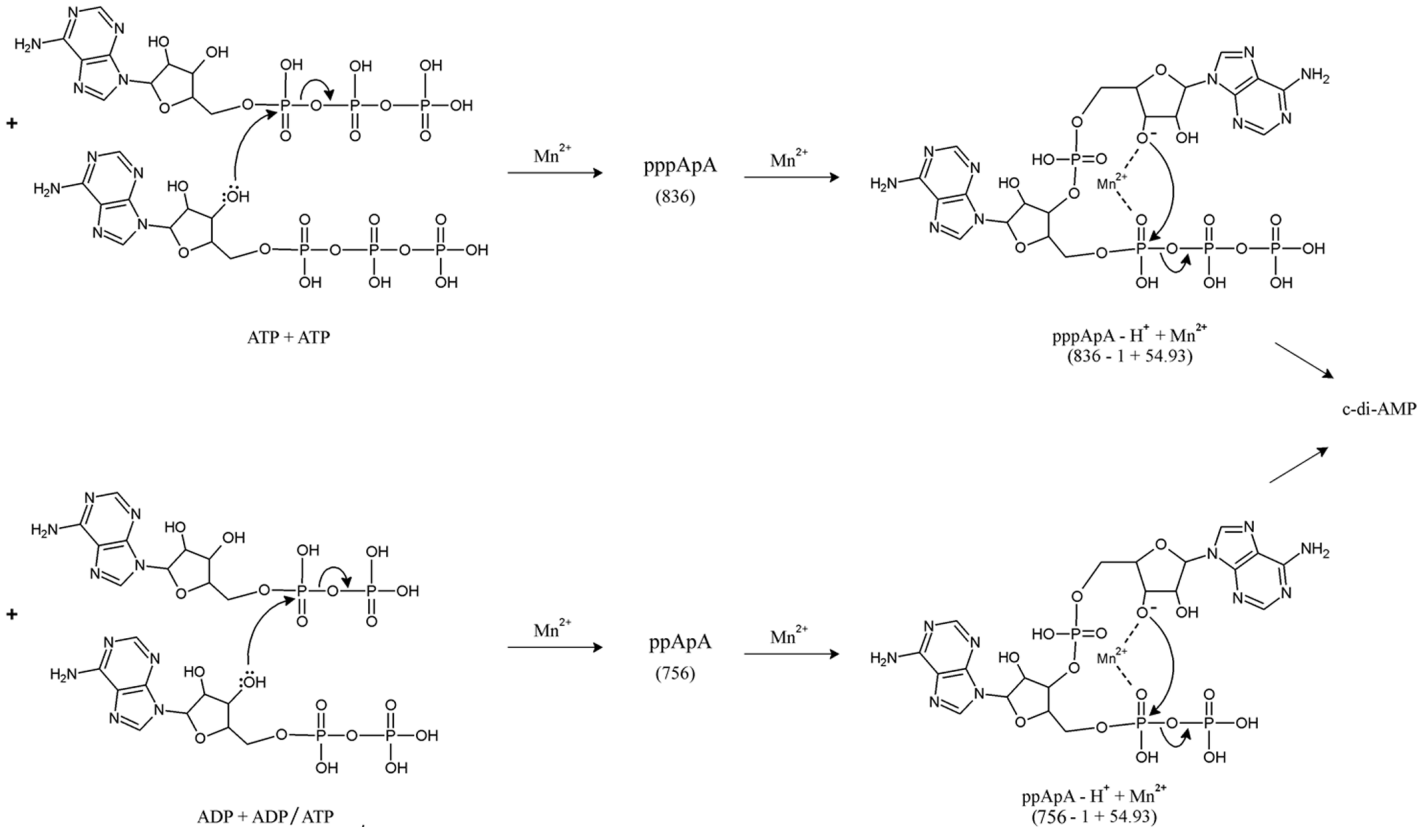
Hydrolysis of c-di-AMP by MtbPDE. The reaction mixtures of PDE assay were separated by reverse phase LC and the products were detected by measuring absorbance at 260–4 min have been shown here. The peaks have been labeled with the name of the eluted species. Number on the peak is the retention time of the species. (A) shows the hydrolysis product of c-di-AMP by MtbPDE. Inset shows the control reaction without the enzyme. (B) pApA was hydrolyzed by MtbPDE to AMP. Inset shows the control reaction without MtbPDE. (C) MtbPDE hydrolyzes ApA to AMP and adenosine (D) Hydrolysis of c-di-GMP by MtbPDE to 5′-GMP. Inset shows the control reaction without MtbPDE. (E) Mutant protein D130AH131A does not hydrolyze c-di-AMP.

To determine the enzymatic activity of Rv2837c (MtbPDE), the recombinant protein was purified from *E*. *coli* and used for enzymatic studies (Figure S1B). MtbPDE has a DHH domain which has been shown to be present in proteins with polyphosphatase or nuclease (phosphodiesterase) activity [25]. It has another domain, DHHA1 at its C-terminus which has conserved amino acid residues ‘GGGH’. To determine the phosphoesterase activity of the protein, we utilized non-DNA substrates, *p*-nitrophenyl phosphate (*p*NPP) and bis *p*-nitrophenyl phosphate (bis-*p*NPP). MtbPDE hydrolyzed bis-*p*NPP to *p*-nitrophenol giving rise to yellow color. Formation of *p*-nitrophenol was monitored spectrophotometrically by measuring the absorbance at 410 nm (Figure S5) [26].The protein did not hydrolyze *p*-nitrophenyl phosphate under similar conditions suggesting that it has only phosphodiesterase (PDE) activity. We then determined the PDE activity of MtbPDE on cyclic nucleotides cAMP, cGMP, c-di-AMP and c-di-GMP. The enzyme did not hydrolyze cAMP and cGMP (data not shown) but it hydrolyzed c-di-AMP and c-di-GMP (Figure 7) to 5′-AMP and 5′-GMP respectively.

The products of the PDE assays containing c-di-AMP as substrate were identified by separating the reaction mixture by reverse phase LC. Two peaks of retention time (RT) 1.42 min and 2.04 min were detected in the chromatogram (Figure 7A). The peaks were identified to be of AMP and pApA respectively. MALDI of the reaction mixture showed peaks corresponding to molecular masses of 676 (linear hydrolysis product of c-di-AMP, pApA) and 347 (AMP) Da (data not shown). The peaks for pApA and AMP were confirmed by comparing their RTs with that of pApA (BioLog) and 5′-AMP. These results suggest that MtbPDE sequentially hydrolyzes the two phosphodiester bonds of c-di-AMP, pApA being the product after the hydrolysis of one of the phosphodiester bonds and 5′-AMP is produced after the hydrolysis of the second phosphodiester bond. The hydrolysis of pApA to AMP is the oligoribonuclease activity of MtbPDE as has been shown before [27]. We confirmed the oligoribonuclease activity of MtbPDE by incubating pApA with the protein. pApA was hydrolyzed to AMP whereas ApA, obtained after treating pApA with alkaline phosphatase was hydrolyzed to 5′-AMP and adenosine (Figure 7B & C) showing that the protein hydrolyzes the phosphodiester bond from 3′-end giving rise to 5′-nucleotide. MtbPDE hydrolyzed c-di-GMP to 5′-GMP (Figure 7D) though c-di-AMP is the preferred substrate. An enzyme titration showed that the turnover number of MtbPDE with c-di-AMP, c-di-GMP and pApA as substrate are 50,1.5 and 30/min respectively (Figure S5).

We produced the mutant MtbPDE protein by mutating D130 and H131 amino acid residues of ‘DHH’ motif to alanine. The resulting protein D130AH131A failed to hydrolyze c-di-AMP suggesting that the motif is involved in hydrolyzing c-di-AMP (Figure 7E).

We also analyzed the RecJ-like nuclease (phosphodiesterase) activity of MtbPDE on single stranded DNA substrate as RecJ also has ‘DHH’ domain. The protein was incubated with a single stranded 24-mer oligonucleotide and the reaction products were separated by reverse phase LC. We did not detect any nuclease or RecJ like activity (data not shown).

## Discussion

Cyclic di-AMP is a recently discovered signaling molecule in eubacteria and there is not much understanding of its signaling network. We have characterized here two proteins of *M*. *tuberculosis* which metabolize c-di-AMP. We have shown that Rv3586 has the DAC activity and can synthesize c-di-AMP from ATP in a two-step process whereas Rv2837c can hydrolyze the two phosphodiester bonds of c-di-AMP sequentially, the first one resulting in linear 5′-phosphate di adenosine nucleotide (pApA) which is further hydrolyzed to 5′-AMP.

Detailed characterization of the DAC activity revealed that the activity was sensitive to the metal ion and ATP (substrate) concentration. We noted a sharp decline in the activity with an increase in the metal ion concentration over the optimum one. We also detected a low ATPase activity of MtbDisA. However, significant ATPase activity was observed in the mutant protein D72AG73A which did not have any DAC activity (Figure 2D). These observations suggest the presence of separate active sites for the DAC and ATPase activities and by preferential binding of the ATP to the DAC active site, ATPase activity is masked in the wild type MtbDisA. More than one binding site for ATP can also be suggested from the fact that higher concentrations of ATP lead to a sharp decrease in the DAC activity. At higher ATP concentrations, the ATP binds to the secondary site (most probably the site for ATPase activity) and allosterically inhibits the DAC activity. Similar inhibition of the DAC activity has been noted when ADP is used as the substrate for the reaction. It appears that the bacteria have evolved different mechanisms to regulate the cellular concentration of c-di-AMP. We have also identified the multimeric forms of c-di-AMP by LC-ESI-MS which adds one more facet to the regulation of this signaling molecule. Dimeric, trimeric and tetrameric forms of c-di-AMP were detected. These different forms of c-di-AMP can be involved in regulating various physiological processes by binding to different types of receptors, by sequestering cellular c-di-AMP concentration etc. It has recently been reported that two structural polymorphic forms of c-di-GMP regulate two different activities in *Xanthomonas campestris* pv. *campestris* by binding to two different types of PilZ-domain protein [23].

The inhibition of the enzyme (MtbDisA) at higher substrate (ATP) concentration might have physiological significance. The cellular concentration of ATP in mycobacterium is 1 mM [28] and MtbDisA is inhibited at this concentration. There is a possibility that in the logarithmic phase of the growth when the concentration of ATP is high, there is no c-di-AMP formation but as the bacteria are under ‘stress’ which leads to decrease in the cellular ATP concentration, c-di-AMP is synthesized. This helps bacteria to survive under stress. Dissecting the different pathways regulated by c-di-AMP will be an important exercise and as c-di-AMP and the pathways regulated by it are specific for bacteria, they could be potential drug targets. There is a strong possibility that c-di-AMP is involved in DNA damage signaling because of the conservation of the operon containing *radA* and *disA* genes in mycobacterium [29]. It is provocative to think that DisA and RadA act together at DNA breaks. Genetic studies have suggested the involvement of RadA in the processing of DNA recombination intermediates, most likely the branched intermediates and restoration of replication fork in *E*. *coli* [30], [31]. It is interesting to note that the operon containing *radA* and *disA* is induced as a part of RecA- independent SOS response [32], [33]. There is a likelihood of c-di-AMP regulating RecA-independent SOS response.

The identification of the intermediates of the DAC reaction suggests a two-step synthesis of c-di-AMP from ATP or ADP (Figure 5). The first step involves the nucleophilic attack by the 3′-hydroxy of ribose sugar of ATP or ADP onto the α-phosphate of opposing ATP or ADP molecule with the release of pyrophosphate or phosphate molecule giving rise to the intermediate pppApA or ppApA respectively. This is followed by the cyclization of these linear intermediates which involves a similar nucleophilic attack by the 3′-hydroxy of the other ribose sugar onto the other α-phosphate resulting in c-di-AMP through the intermediate ‘II’. The intermediate ‘II’ is a Mn^2+^- complex (transition state complex) of the deprotonated intermediates pppApA or ppApA. 3′-OH group of the ribose sugar of the two intermediates is deprotonated in the Mn^2+^-adduct (as shown in Figure 5) which facilitates the nucleophilic attack on the α-phosphate leading to the formation of c-di-AMP. Intermediate ppApA can also arise by condensation between ADP and ATP molecules as shown in Figure 5. Conversion of ADP into c-di-AMP is extremely poor (Figure 2C) and is not the optimal substrate for MtbDisA. Similar two-step synthesis has been proposed recently for the synthesis of cyclic-GMP-AMP (cGAMP) by cGAMP synthase, cGAS [34].

The DAC activity of MtbDisA has been reported before [21] but we have not been able to get any activity under the conditions reported. We have rather found that 2 mM of Mn^2+^ and ATP are inhibitory for the activity. We have determined the optimum concentration of Mn^2+^ and ATP to be 0.6 and 0.3 mM respectively by systematic titration of both of them. We have also been not able to detect AMP and pApA in our DAC reaction mixture.

We have identified Rv2837c (MtbPDE) as the protein which hydrolyzes c-di-AMP. Rv2837c is a soluble protein and has the core DHH/DHHA1 domain. ‘DHH’ domain has been shown to be involved in phosphodiesterase activity as in *E*. *coli* RecJ nuclease [25] whereas ‘DHHA1’ has been suggested to be involved in the substrate specificity of YybT (or GdpP) or its orthologs [8], [14], [24]. MtbPDE does not have any RecJ-like nuclease activity but it hydrolyzes sequentially the two phosphodiester bonds of c-di-AMP. c-di-AMP is first hydrolyzed to linear 5′-phosphorylated diadenosine nucleotide (pApA) which is further hydrolyzed to 5′-AMP. The hydrolysis of the linear diadenosine nucleotide (pApA) to AMP is in accordance with the nanoRNase activity of Rv2837c [27]. MtbPDE is specific for hydrolyzing c-di-AMP and does not hydrolyze other cyclic nucleotides, cAMP or cGMP though there is little hydrolysis of c-di-GMP to 5′-GMP. MtbPDE hydrolyzes linear pApA substrate to AMP whereas ApA dinuceotide is hydrolyzed to AMP and adenosine. The hydrolysis products suggest that the enzyme attacks the substrate from the 3′ end with the production of 5′-ribonucleotides [35]. NanoRNAs have been shown recently to be involved in transcription initiation and selection of transcription initiation site, gene expression [36], [37] and there is a possibility that c-di-AMP regulate these processes.

MtbPDE is a conserved protein and its homologs are present across the bacterial kingdom including *Bacillus subtilis* which already has YybT. Homologs of MtbPDE in *B*. *subtilis* are NrnA and NrnB which have been shown to have the nanoRNase activity and have conserved DHH/DHHA1 domain [38]. The oligoribonuclease protein, Orn from *E*. *coli* and *M*. *tuberculosis* (Rv2511) are smaller proteins of 181 and 215 amino acid residues respectively and do not have DHH/DHHA1 domain. Proteins with DHH/DHHA1 domain appear to have evolved as c-di-AMP specific phosphodiesterase and also retaining the oligoribonuclease activity. This new activity of ‘DHH/DHHA1’ domain containing proteins will expand the role of c-di-AMP signaling network. MgpA and P1 proteins from *M*. *genitalium* and *M*. *pneumoniae* respectively containing DHH/DHHA1 domains exist in an operon containing cytoadherence-associated proteins [25] and P1 has been shown to be involved in *M*. *pneumoniae* cytoadherence [39]. Recently, a c-di-AMP specific phosphodiesterase, SPD_1153 has been reported from *S*. *pneumoniae* [40]. This protein consists of an open reading frame of 311 amino acid residues and is a soluble protein like MtbPDE. The protein contains DHH/DHHA1 domain and hydrolyzes c-di-AMP and pApA to AMP though hydrolysis of c-di-AMP to pApA has not been observed.

Rv2837c (MtbPDE) has also been shown to have phosphatase activity on pAp, a product of sulfur metabolism and secondary metabolites like polyketide synthesis [27]. The phosphatase activity on pAp is phosphomonoesterase activity and it is possible that MtbPDE has evolved as phosphoesterase (both mono and diesterase) on different substrates under specific conditions. Multiple activities of this protein make it essential for the survival of mycobacterium [41]. It will be interesting to determine how the different activities are regulated *in vivo*.

## Supporting Information

Figure S1 **MtbDisA and MtbPDE proteins.** (A) Schematic diagrams of MtbDisA and MtbPDE depicting the different domains present in the proteins. (B)Purified wild type and mutant MtbDisA and MtbPDE recombinant proteins: 7.5 µg of the purified wild type and mutant MtbDisA and MtbPDE proteins as indicated at the top of the lane were analyzed by 12% SDS-PAGE. The gel was stained with Coomassie blue dye and has been shown here. The sizes (kDa) and positions of molecular weight markers have been indicated on the right.
(TIF)

Figure S2 **c-di-AMP is formed in the DAC reaction.** The DAC reaction mixture was subjected to LC-ESI-MS. (A)ESI-MS/MS spectrum of precursor ion m/z 659.11. (B) Scheme delineating the MS/MS spectrum in ‘A’ deciphering the molecule to be c-di-AMP.
(TIF)

Figure S3 **Characterization of the manganese adduct of the intermediate ‘I’.** (A) LC - ESI - MS/MS spectra of singly charged ([M+Mn^2+^ - H^+^]; m/z 889.98) and doubly charged ([M+Mn^2+^]; m/z 445.49) precursor ions. (B) Schemes depicting the interpretation of fragmentation as noted from (A), (B) for [M+Mn^2+^ - H^+^]; precursor m/z 889.98; (C) for [M+Mn^2+^]; precursor m/z 445.49.
(TIF)

Figure S4 **ppApA is another intermediate of the DAC reaction.** (A) MALDI-MS/MS spectrum of [M+H]^+^ precursor ion m/z 757.11. (B) Scheme providing the interpretation of MS/MS spectrum in ‘A’ leading to the identification of molecular structure of the intermediate.
(TIF)

Figure S5 **MtbPDE has phosphodiesterase activity.** (A) The hydrolysis of bis-*p*-nitrophenyl phosphate and *p*-nitrophenyl phosphate to *p*-nitrophenol was determined in 20 µl reaction mixtures containing 50 mM Tris-HCl (pH 8.0), 5 mM bis-*p*NPP or *p*NPP, 0.1 µM MtbPDE and 2.5 mM MnCl_2_. The reaction was incubated at 37°C for 15 min. Amount of *p*-nitrophenol formed has been shown. (B) Metal ion dependence of the hydrolysis of c-di-AMP by MtbPDE: Hydrolysis of c-di-AMP was carried out in 10 µl reaction mixtures containing 50 mM Tris-HCl (pH 8.0), 5.0 mM of different divalent metal ion, 1 mM DTT, 100 µM c-di-AMP and 0.1 µM MtbPDE. The reaction mixtures were incubated for 10 min at 37°C. Amount of c-di-AMP hydrolyzed has been shown (mean of three independent experiments ± standard deviation). (C) &(D) Reaction mixtures (10 µl) containing 50 mM Tris-HCl (pH 8.0), 5 mM MnCl_2_, 1 mM DTT, 100 µM c-di-AMP or c-di-GMP and the indicated amounts of MtbPDE were incubated at 37°C for 10 mins. The amount of c-di-AMP/c-di-GMP hydrolyzed has been plotted as a function of the amount of MtbPDE in the reaction mixture.
(TIF)
