# Correction: Two-Step Synthesis and Hydrolysis of Cyclic di-AMP in *Mycobacterium tuberculosis*

**DOI:** 10.1371/journal.pone.0096590

**Published:** 2014-04-24

**Authors:** The PLOS ONE Staff

Figures 5, 6, 7, and S4 are incorrect. The authors have provided corrected versions here.

**Figure 5:**
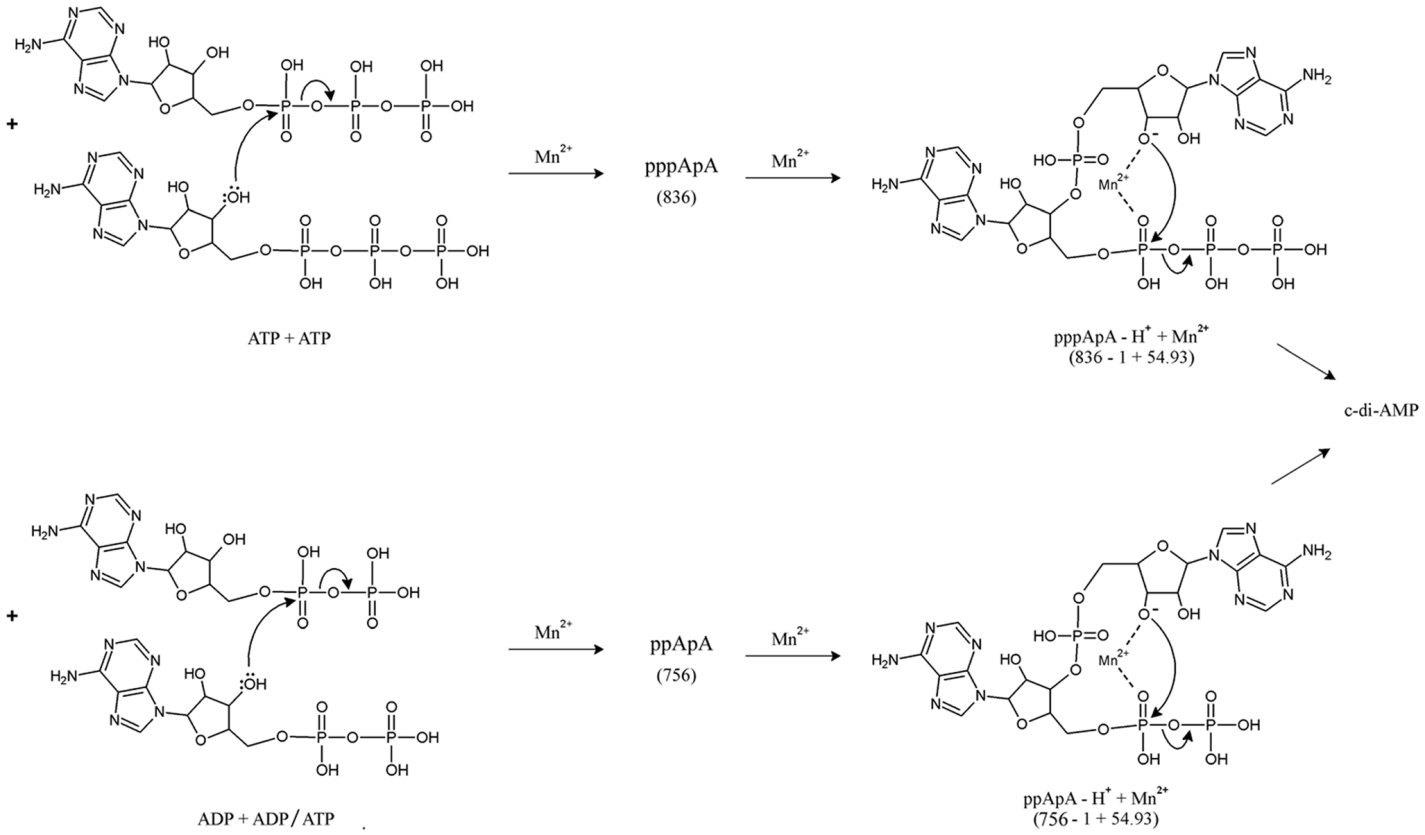
Two-step synthesis of c-di-AMP. The figure shows the formation of c-di-AMP from the two intermediates pppApA and ppApA. pppApA is first formed by conjugation between two molecules of ATP (step 1A). Two ADP molecules or one molecule of ADP and one ATP molecule can conjugate to give rise to ppApA (step 2A). pppApA and ppApA are then converted into c-di-AMP (steps 1B & 2B) through their respective Mn^2+^-adduct (transition state complex) as shown.

**Figure 6:**
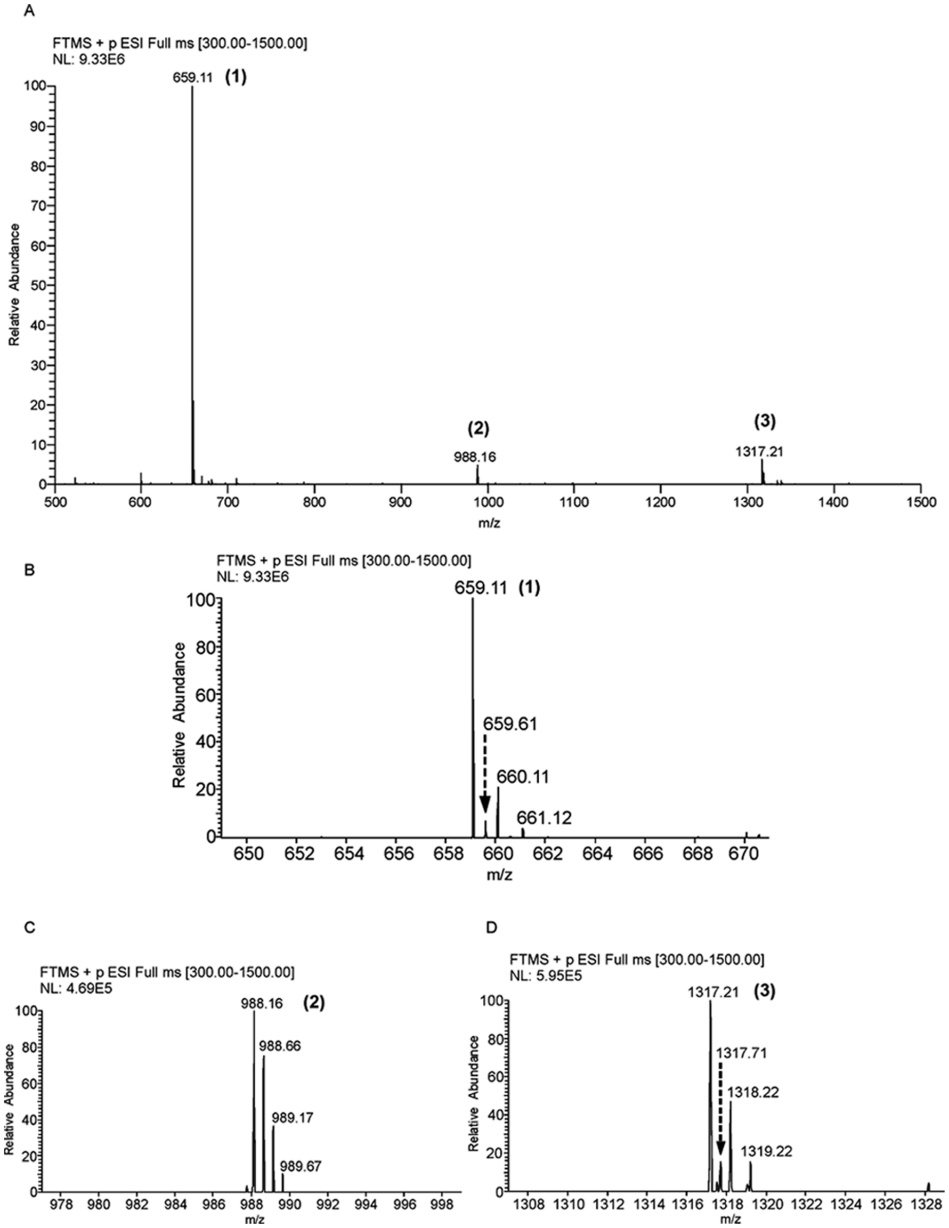
c-di-AMP can exist as multimers. The DAC reaction mixture was subjected to LC-ESI-MS and the mass spectra of the different multimeric forms have been shown here. (A) The LC-ESI mass spectrum showing signals corresponding to monomer, labeled ‘1′ and other multimeric forms, labeled ‘2′ and ‘3′ (B) Expansion of the region, m/z 648–672 of the spectrum in ‘A’ depicting isotope peaks corresponding to peak 1, which indicate presence of monomer and dimer (C) Expansion of the region, m/z 976–1000 of the spectrum in ‘A’, which shows isotope signals corresponding to peak 2 providing evidence for the presence of trimer of c-di-AMP (D) Expansion of the region, m/z 1306–1330 of the spectrum in ‘A’, indicating the presence of dimeric and tetrameric forms of c-di-AMP.

**Figure 7:**
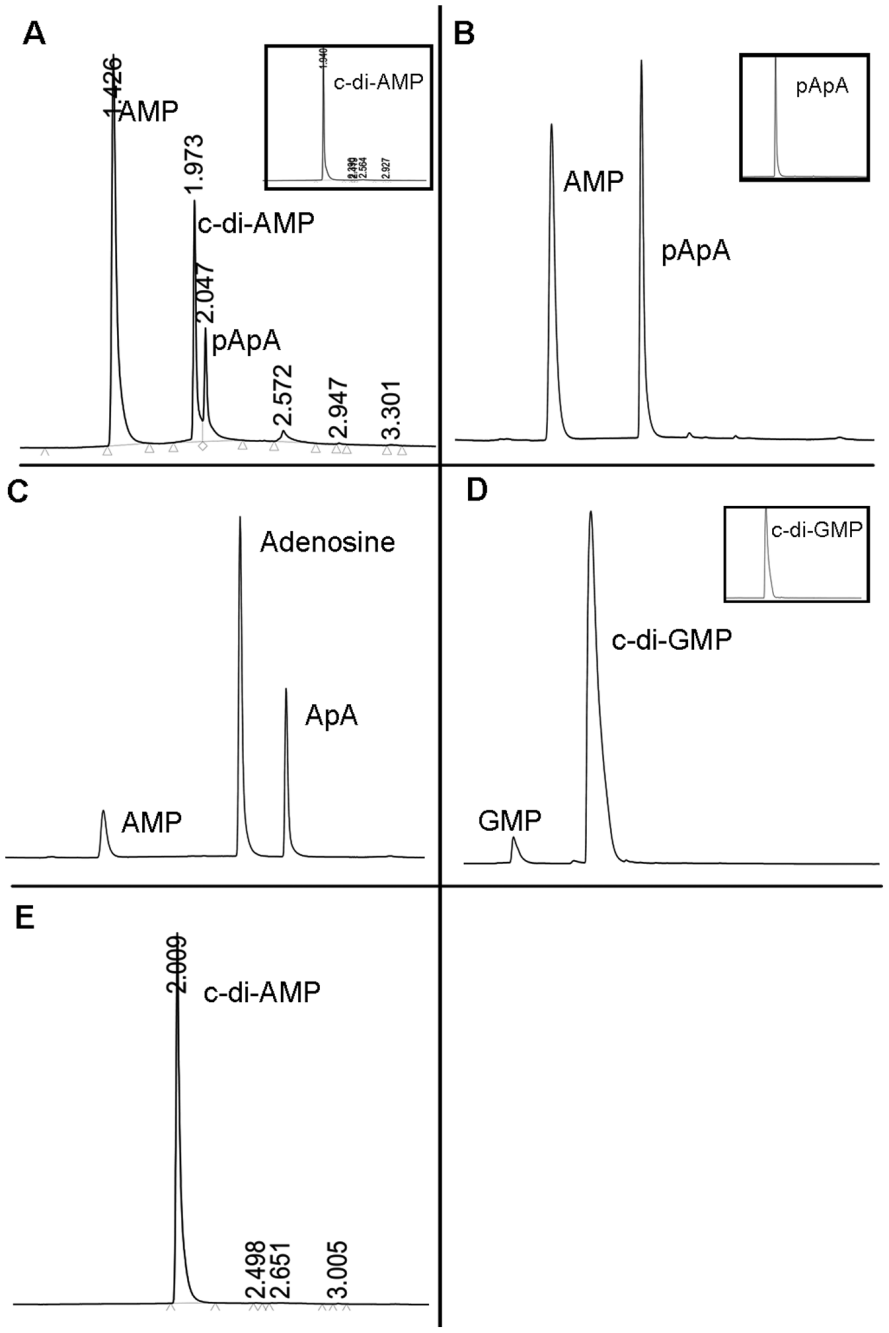
Hydrolysis of c-di-AMP by MtbPDE. The reaction mixtures of PDE assay were separated by reverse phase LC and the products were detected by measuring absorbance at 260–4 min have been shown here. The peaks have been labeled with the name of the eluted species. Number on the peak is the retention time of the species. (A) shows the hydrolysis product of c-di-AMP by MtbPDE. Inset shows the control reaction without the enzyme. (B) pApA was hydrolyzed by MtbPDE to AMP. Inset shows the control reaction without MtbPDE. (C) MtbPDE hydrolyzes ApA to AMP and adenosine (D) Hydrolysis of c-di-GMP by MtbPDE to 5′-GMP. Inset shows the control reaction without MtbPDE. (E) Mutant protein D130AH131A does not hydrolyze c-di-AMP.

## Supporting Information

Figure S4 ppApA is another intermediate of the DAC reaction. (A) MALDI-MS/MS spectrum of [M+H]^+^ precursor ion m/z 757.11. (B) Scheme providing the interpretation of MS/MS spectrum in ‘A’ leading to the identification of molecular structure of the intermediate.
(TIF)
