# Supplementary figures and images for: Two-Step Synthesis and Hydrolysis of Cyclic di-AMP in Mycobacterium tuberculosis
Source: PLoS One. 2014 Jan 23;9(1):e86096. doi: 10.1371/journal.pone.0086096 (PMC3900455; doi:10.1371/journal.pone.0086096)

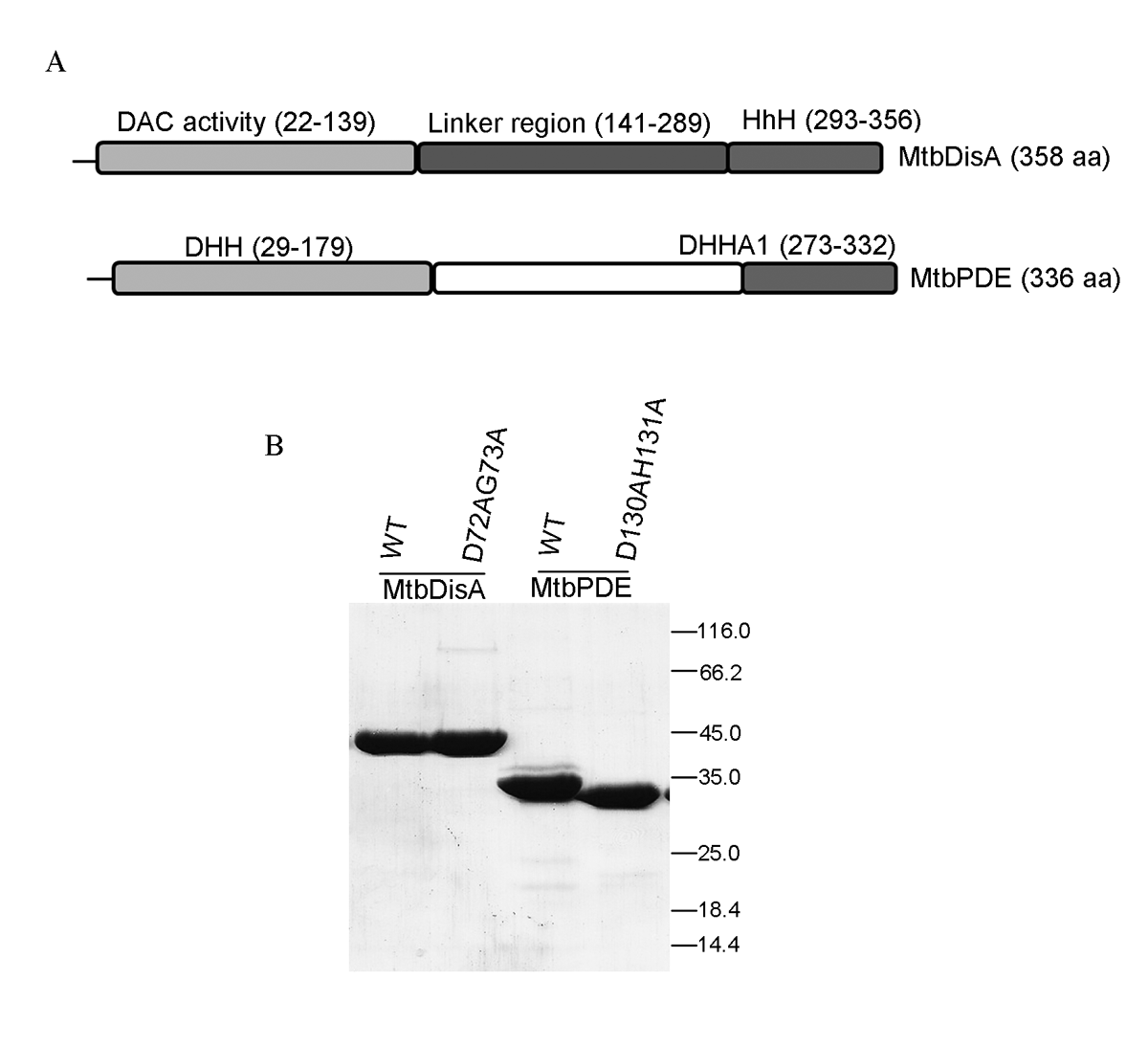

Supplement: Figure S1 — MtbDisA and MtbPDE proteins. (A) Schematic diagrams of MtbDisA and MtbPDE depicting the different domains present in the proteins. (B)Purified wild type and mutant MtbDisA and MtbPDE recombinant proteins: 7.5 µg of the purified wild type and mutant MtbDisA and MtbPDE proteins as indicated at the top of the lane were analyzed by 12% SDS-PAGE. The gel was stained with Coomassie blue dye and has been shown here. The sizes (kDa) and positions of molecular weight markers have been indicated on the right. (TIF) [file pone.0086096.s001.tif]

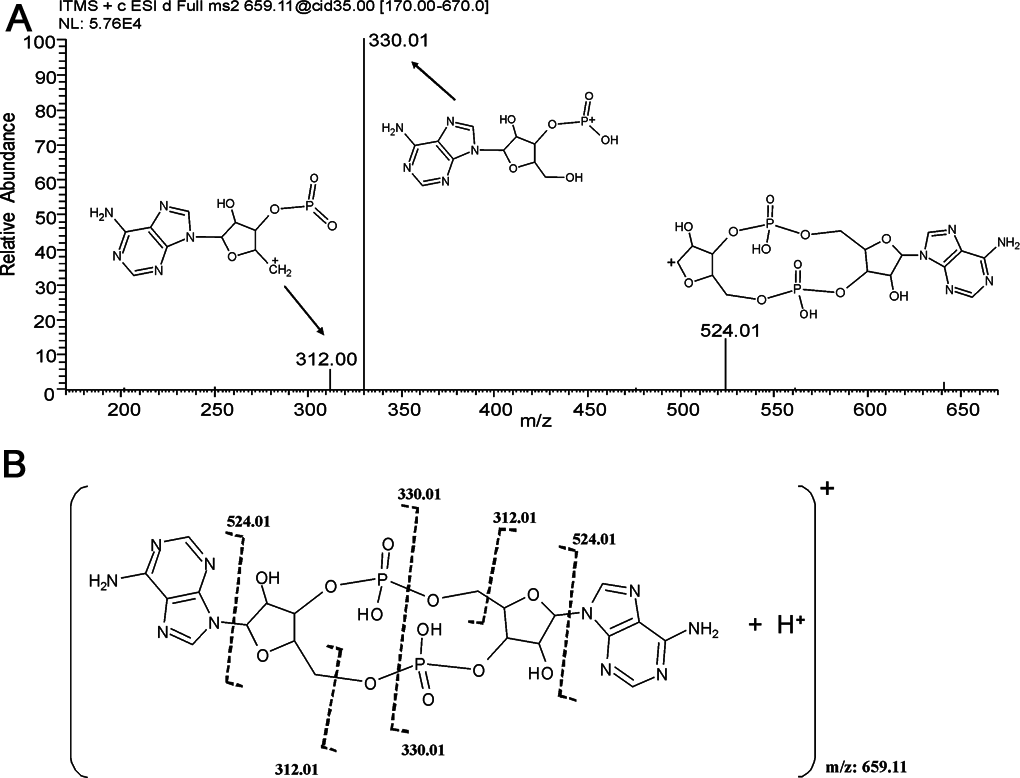

Supplement: Figure S2 — c-di-AMP is formed in the DAC reaction. The DAC reaction mixture was subjected to LC-ESI-MS. (A)ESI-MS/MS spectrum of precursor ion m/z 659.11. (B) Scheme delineating the MS/MS spectrum in ‘A’ deciphering the molecule to be c-di-AMP. (TIF) [file pone.0086096.s002.tif]

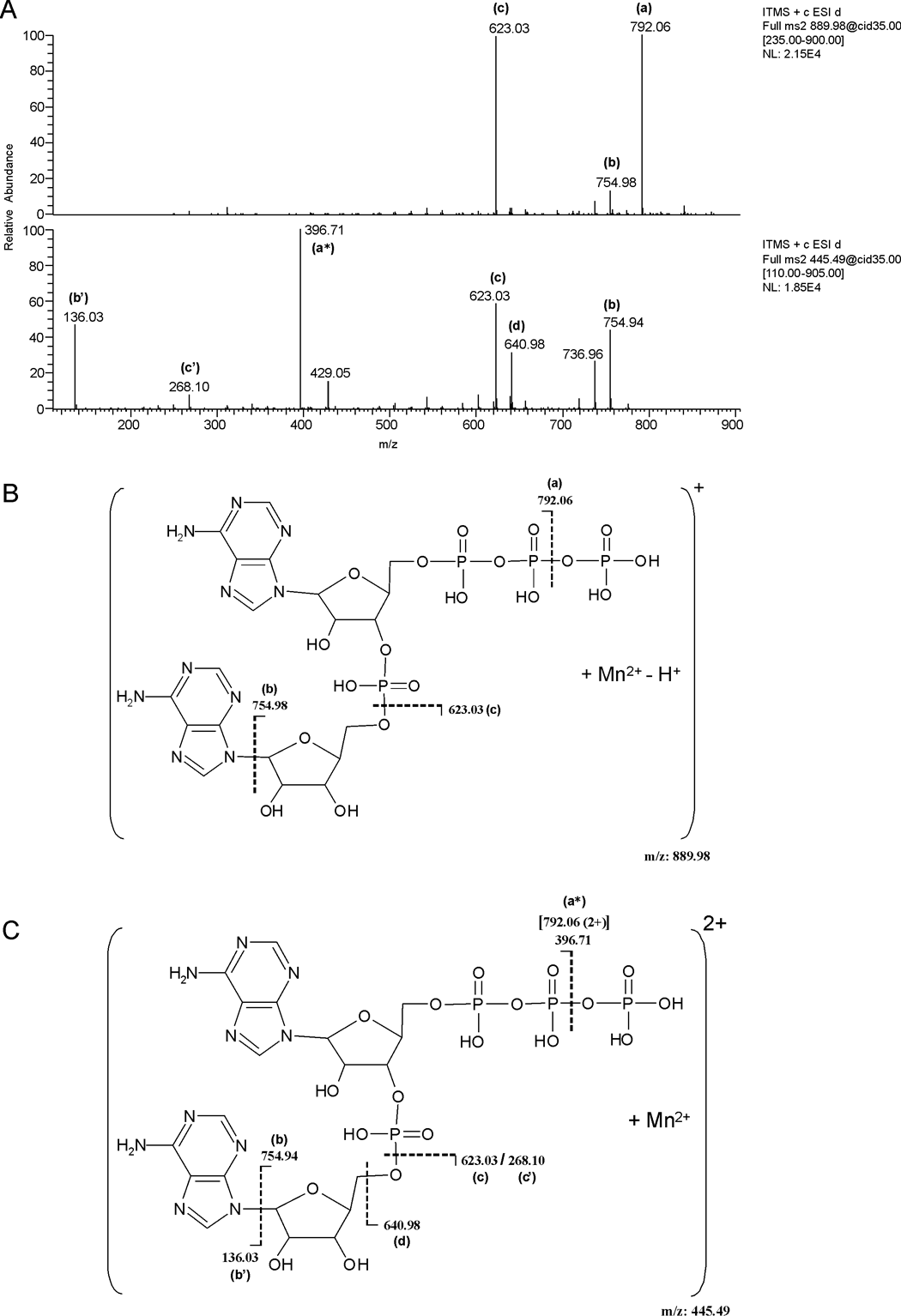

Supplement: Figure S3 — Characterization of the manganese adduct of the intermediate ‘I’. (A) LC - ESI - MS/MS spectra of singly charged ([M+Mn2+ - H+]; m/z 889.98) and doubly charged ([M+Mn2+]; m/z 445.49) precursor ions. (B) Schemes depicting the interpretation of fragmentation as noted from (A), (B) for [M+Mn2+ - H+]; precursor m/z 889.98; (C) for [M+Mn2+]; precursor m/z 445.49. (TIF) [file pone.0086096.s003.tif]

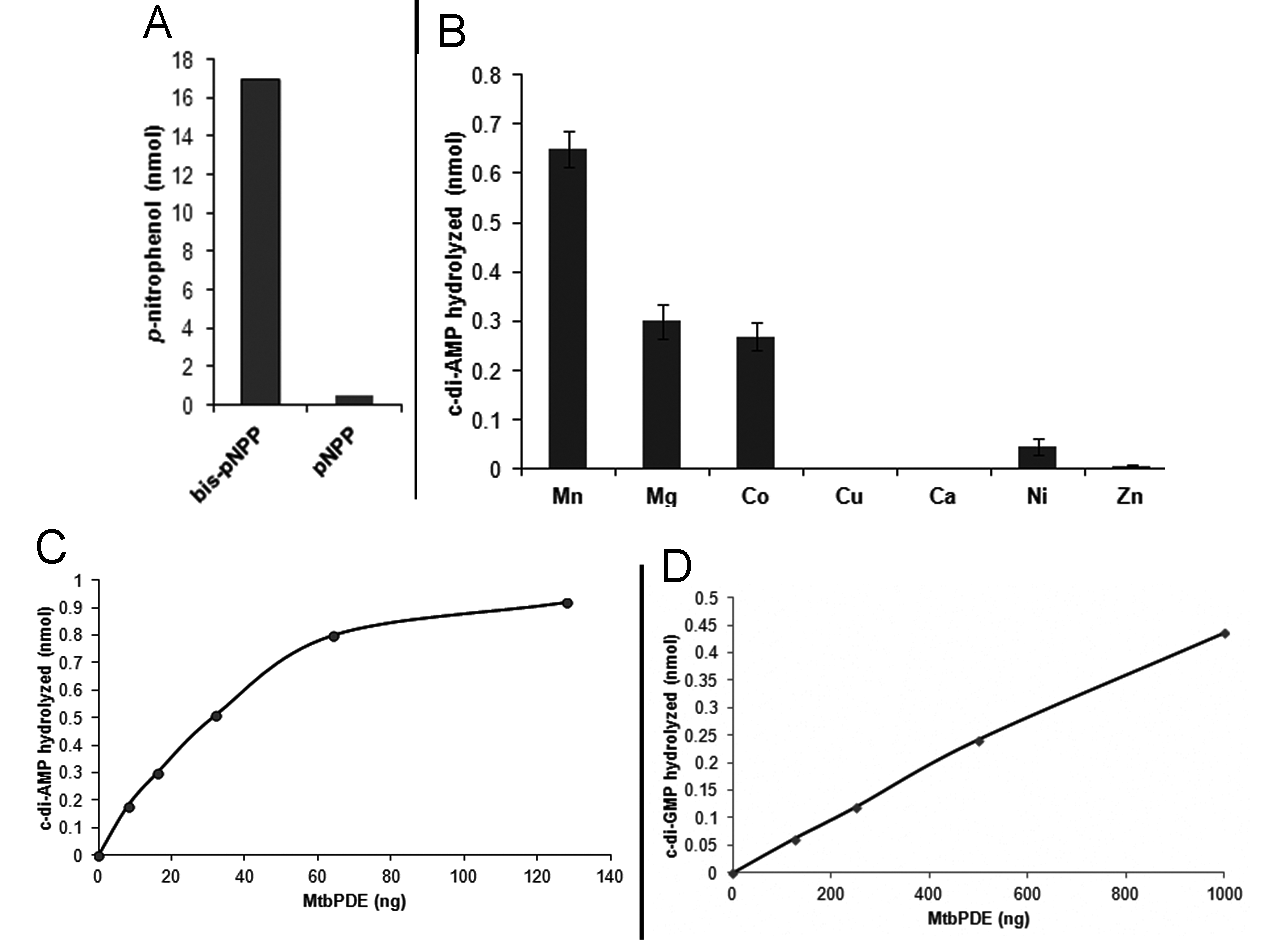

Supplement: Figure S5 — MtbPDE has phosphodiesterase activity. (A) The hydrolysis of bis-p-nitrophenyl phosphate and p-nitrophenyl phosphate to p-nitrophenol was determined in 20 µl reaction mixtures containing 50 mM Tris-HCl (pH 8.0), 5 mM bis-pNPP or pNPP, 0.1 µM MtbPDE and 2.5 mM MnCl2. The reaction was incubated at 37°C for 15 min. Amount of p-nitrophenol formed has been shown. (B) Metal ion dependence of the hydrolysis of c-di-AMP by MtbPDE: Hydrolysis of c-di-AMP was carried out in 10 µl reaction mixtures containing 50 mM Tris-HCl (pH 8.0), 5.0 mM of different divalent metal ion, 1 mM DTT, 100 µM c-di-AMP and 0.1 µM MtbPDE. The reaction mixtures were incubated for 10 min at 37°C. Amount of c-di-AMP hydrolyzed has been shown (mean of three independent experiments ± standard deviation). (C) &(D) Reaction mixtures (10 µl) containing 50 mM Tris-HCl (pH 8.0), 5 mM MnCl2, 1 mM DTT, 100 µM c-di-AMP or c-di-GMP and the indicated amounts of MtbPDE were incubated at 37°C for 10 mins. The amount of c-di-AMP/c-di-GMP hydrolyzed has been plotted as a function of the amount of MtbPDE in the reaction mixture. (TIF) [file pone.0086096.s005.tif]
