# Supplementary figures and images for: Correction: Two-Step Synthesis and Hydrolysis of Cyclic di-AMP in Mycobacterium tuberculosis
Source: PLoS One. 2014 Apr 24;9(4):e96590. doi: 10.1371/journal.pone.0096590 (PMC3999191; doi:10.1371/journal.pone.0096590)

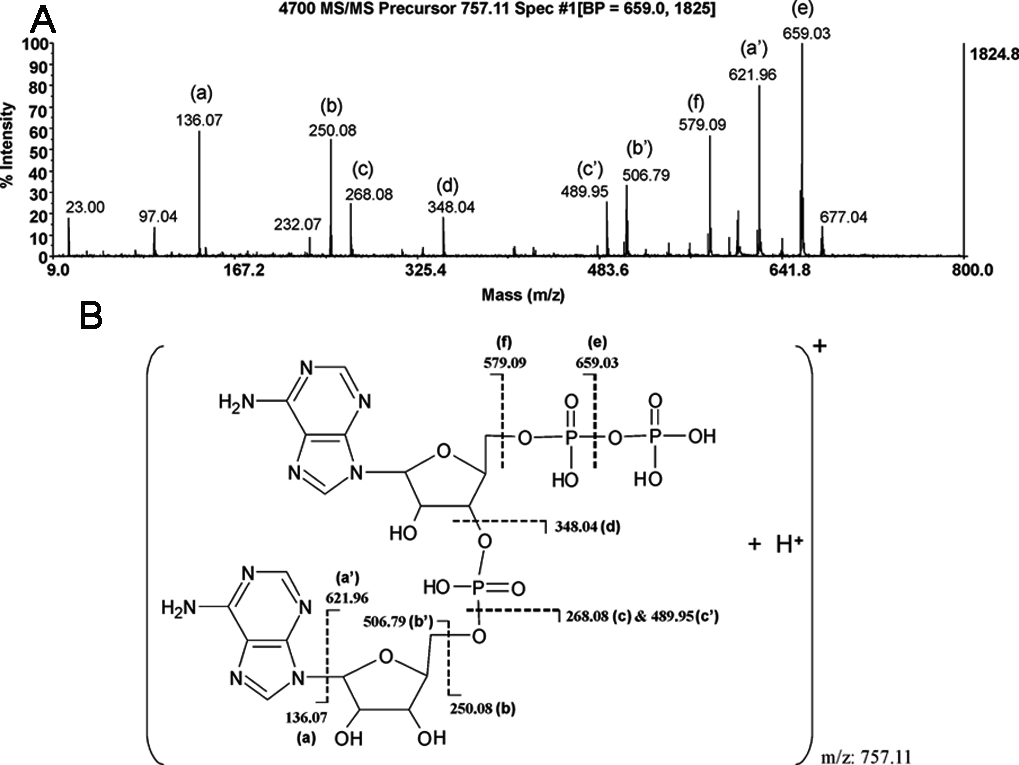

Supplement: Figure S4 — ppApA is another intermediate of the DAC reaction. (A) MALDI-MS/MS spectrum of [M+H]+ precursor ion m/z 757.11. (B) Scheme providing the interpretation of MS/MS spectrum in ‘A’ leading to the identification of molecular structure of the intermediate. (TIF) [file pone.0096590.s001.tif]
